# Factors Influencing the Frequency of Airway Infections in Underage Refugees: A Retrospective, Cross Sectional Study

**DOI:** 10.3390/ijerph17186823

**Published:** 2020-09-18

**Authors:** Frank Müller, Eva Hummers, Nele Hillermann, Christian Dopfer, Alexandra Jablonka, Tim Friede, Anne Simmenroth, Martin Wetzke

**Affiliations:** 1 Department of General Practice, University Medical Center Goettingen, 37073 Goettingen, Germany; eva.hummers@med.uni-goettingen.de (E.H.); nele.hillermann@stud.uni-goettingen.de (N.H.); 2 Department of Pediatrics, Neonatology and Allergology, Hannover Medical School, 30625 Hannover, Germany; dopfer.christian@mh-hannover.de (C.D.); wetzke.martin@mh-hannover.de (M.W.); 3 German Center for Infection Research (DZIF), partner site Hannover-Braunschweig, 38124 Braunschweig, Germany; jablonka.alexandra@mh-hannover.de; 4 Department of Rheumatology and Immunology, Hannover Medical School, 30625 Hannover, Germany; 5 Department of Medical Statistics, University Medical Center Goettingen, 37073 Goettingen, Germany; tim.friede@med.uni-goettingen.de; 6 Department of General Practice, University Hospital Wuerzburg, 97080 Wuerzburg, Germany; simmenroth_a@ukw.de

**Keywords:** refugees, migrants, respiratory infection, children, adolescents, seasonality, crowded housing, COVID-19, containment

## Abstract

*Background*: Infections are a leading cause of refugee morbidity. Recent data on the rate of airway infections and factors influencing their spread in refugee reception centers is scarce. *Methods*: A retrospective, cross-sectional study of de-identified medical records with a focus on respiratory infections in underage refugees was conducted at two large German refugee reception centers. *Results*: In total, medical data from *n* = 10,431 refugees over an observational period of *n* = 819 days was analyzed. Among pediatric patients (*n* = 4289), 55.3% presented at least once to the on-site medical ward with an acute respiratory infection or signs thereof. In 38.4% of pediatric consultations, acute airway infections or signs thereof were present. Airway infections spiked during colder months and were significantly more prevalent amongst preschool and resettled children. Their frequency displayed a positive correlation with the number of refugees housed at the reception centers. *Conclusions*: We show that respiratory infections are a leading cause for morbidity in young refugees and that their rate is influenced age, season, status, and residential density. This illustrates the need to protect refugee children from contracting airway infections which may also reduce the spread of coronavirus disease 2019 (COVID-19) during the current pandemic.

## 1. Background

Refugee children have been a population particularly prone to communicable diseases throughout history [1,2,3,4]. Although children and adolescents belong to the most vulnerable population in the current refugee crisis, data on health concerns in this population is scarce [5]. Previous studies reported on the increased infection susceptibility in underage refugees during the current refugee crisis [6,7]. Here, we sought to analyze data of our large record on primary healthcare in migrants with a particular focus on the prevalence of respiratory infections in underage refugees. This data from a time period directly before onset of the coronavirus disease 2019 (COVID-19) pandemic illustrates that respiratory infections are the primary cause for acute morbidity in young refugees and shows that age, season, and living circumstances significantly influence the rate of airway infections amongst refugee children and adolescents. Our report may raise awareness for this particularly vulnerable group and inform decision making to limit the spread of airway infections amongst refugees and beyond.

## 2. Methods

### 2.1. Study Population

Data on primary medical care provision from two migrant reception centers in Celle and Friedland, Germany were included into this analysis. The study cohorts consisted of *n* = 3104 refugees housed in Celle between September 2015 and June 2016 (parts of this cohort have been previously described [8]) and *n* = 7327 refugees housed in Friedland between August 2017 and January 2019 (parts of this cohort were previously described [9]). Refugees were accommodated in these reception facilities for a transitional period of a few weeks up to several months. Detailed data on the living situation at both sites can be found in two previous studies [6,10]. In brief, refugees were housed in small houses (Friedland) or tents and small houses (Celle) with two to six inhabitants per unit and a maximum regular housing capacity of *n* = 850 inhabitants in Celle and *n* = 820 inhabitants in Friedland. Communal facilities included dining areas, childcare and schooling facilities, and showers/restrooms. At both sites, refugees received on-site meals and support from social services, and day-care and schooling was provided for children of all age groups. Furthermore, on-site medical wards provided primary care to all inhabitants of the facilities which were staffed by emergency medical personnel 24 h per day, supplemented by consultation hours by medical doctors on each workday.

### 2.2. Data Collection and Management

All data in this cross-sectional investigation was depersonalized. Retrospective chart reviews of International Statistical Classification of Diseases and Related Health Problems Version 10 (ICD-10) coded diagnoses and signs and symptoms were performed, and for each patient and consultation, information on date of presentation, diagnosis and symptoms, age, sex, and country of origin were collected. Unless refugee children were stateless or unknown, their origin was described according to the system of the World Bank Group [11]. In the study by Kleinert et al. [9] a more detailed description of our approach to ICD-10 based description of refugee healthcare needs can be found. Local authorities provided data on daily facility occupancies which were standardized to the size of respective facilities and are reported as “residential density” in this analysis.

### 2.3. Definitions

Acute respiratory infections were defined as all ICD-10 codings of acute respiratory infections or clear symptoms thereof including cold, cough, sore throat, acute sinusitis, acute pharyngitis, acute tonsillitis, acute laryngitis and tracheitis, influenza, acute bronchitis, and acute bronchiolitis. Acute respiratory infections plus fever was defined as described above but with fever present. Diagnoses such as chronic bronchitis, chronic obstructive pulmonary disease, or vasomotor and allergic rhinopathy were considered as chronic or allergic respiratory diseases and not included into this analysis. Children and adolescents were defined as refugees ≤ 18 years of age. According to their status, refugees were divided into two groups: asylum seeker refugees and resettlement refugees [9]. These two groups differ significantly in their escape conditions and their legal status with the latter group being brought to Germany through a dedicated United Nations High Commissioner for Refugees (UNHCR) resettlement program which is a joint effort by Germany, other European countries such as United Kingdom, Sweden, and France, as well as the United States, Canada, and Australia. Aim of the program is to permanently bring migrants from countries of interim refuge to humanitarian reception in third nations. Before arrival at the new hosting country, resettlement refugees have already gone through the UNHCR refugee-status determination process and therefore do not need to apply for asylum there.

### 2.4. Statistical Analyses

Statistical analyses were performed using SPSS (Version 25, IBM, Armonk, NY, USA), and figures were plotted using GraphPad Prism Version 5 (GraphPad Software Inc., Washington, DC, USA). To describe the frequency of respiratory diseases in relation to sociodemographic factors, we used descriptive statistics including absolute and relative frequencies as well as mean and standard deviation (SD) for categorical and continuous variables, respectively. In addition to these descriptive statistics on consultation level, we also characterized the pediatric population by summarizing data on individual patient level. We compared pediatric patients with and without any respiratory disease in terms of age and frequency of consultations (both analyzed as metric variables) using the nonparametric Mann-Whitney-*U* test (also known as Wilcoxon rank sum test). In order to determine factors that affect the likelihood of a diagnosis of respiratory disease in this setting, we conducted multivariable analyses using logistic regression models. By using generalized estimating equations (GEE) we could correct the standard errors of the regression coefficients and taking into account that patients can have several consultations. *p*-values (*p*) < 0.05 were considered significant.

Power and sample size calculations were carried out with nQuery Advisor version 8.6.0. A sample size of about *n* = 2000 patients was determined to be sufficiently large to detect relevant effect sizes in this population. For instance, the total sample size split in a 4:1 ratio (i.e., *n* = 1600 vs. *n* = 400 patients) provides a power of 95% at a two-sided significance level of 5% given a group difference in airway infections of 50% vs. 40%.

### 2.5. Ethics Compliance

This study was approved by local authorities (Institutional Review Board of Hannover Medical School approval No. 3217–2016, Research Ethics Board of the University Medical Center Goettingen approval No. 16/3/17).

## 3. Results

**Figure 1:**
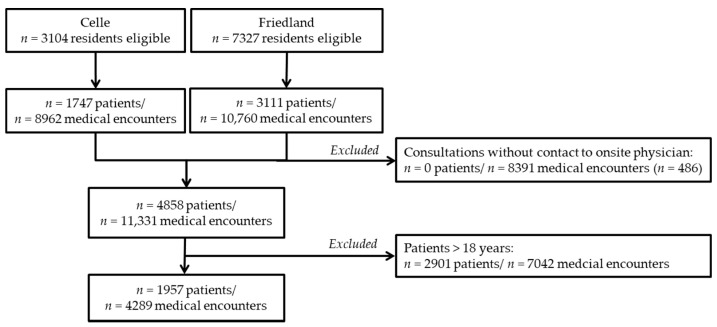
Chart illustrating analysis flow and number of patients and medical encounters included into calculations.

During a total of *n* = 819 days and observational periods encompassing all four seasons at both sites, health care utilization data *n* = 10,431 refugees was recorded. Figure 1 illustrates the analytical flow. Overall, 29.8% (*n* = 4858) of refugees sought on-site medical help at least once during inhabitancy at the reception centers. The mean age of all patients was 24 years (SD 17 years, range 0–81 years, 49.6% female), and 40.3% (*n* = 1957) of them were children and adolescents.

The mean age of pediatric patients was 7.3 years (SD 5.5 years, range 0–18 years), and 46.2% of them were female (Supplementary Table S1). Two thirds (66.5%) of them came from the World Bank Region of the Middle East and North Africa, followed by patients from South Asia (15.1%, Supplementary Table S1). The most prevalent countries of origin were Syria (47.2%), Iraq (15%), and Afghanistan (14.8%, Supplementary Table S1).

**Figure 2:**
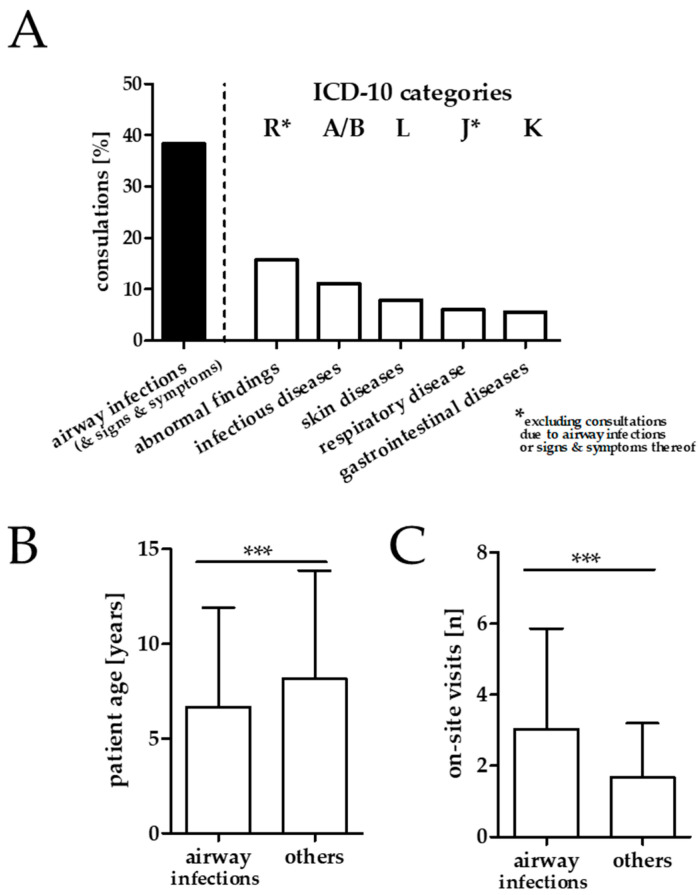
Reasons for on-site medical consultations in underage refugees and age and visit frequency in children with airway infections or signs and symptoms thereof. (**A**): Percentage of consultations due to airway infections or signs and symptoms thereof compared to the percentage of remaining consultations due to complains from the five most frequent ICD-10 code groups. (**B**): The age of children and adolescents with respiratory infections or signs and symptoms thereof. (**C**): The number of on-site visits of children and adolescents with respiratory infections or signs and symptoms thereof. (Note: *: consultations due to airway infections or related symptoms were subtracted from those categorized to group “R”: Symptoms, signs and abnormal clinical and laboratory findings, not elsewhere classified and “J”: Diseases of the respiratory system. “A/B”: certain infectious and parasitic diseases. “L”: diseases of the skin and subcutaneous tissue. “J”: diseases of the respiratory system. “K”: diseases of the digestive system. *** *p* ≤ 0.001.

Acute respiratory infections and symptoms or signs thereof were the most frequent reason for underage refugees seeking on-site medical help. In total, 55.3% of all (*n* = 1957) pediatric and adolescent patients presented at least once with a respiratory infection or signs and symptoms thereof. In total, 38.4% of all consultations in underage refugees occurred due to acute respiratory infections or related symptoms and 7.5% of all pediatric consultations a respiratory infection with fever was treated. As shown in Figure 2A, consultations with airway infections or signs and symptoms occurred more frequently than any of the ICD-10 based diagnose groups in all remaining cases. The top five categories amongst remaining consultations were “R”: Symptoms, signs and abnormal clinical and laboratory findings, not elsewhere classified (15.7%), “A/B”: certain infectious and parasitic diseases (11.1%), “L”: diseases of the skin and subcutaneous tissue (7.9%), “J”: diseases of the respiratory system (6.1%), “K”: diseases of the digestive system (5.5%, Figure 2A). The most common airway infection related ICD-10 single items coded in the ICD-10 system were cough (R05, 12.5%), cold (J00, 8.3%), unspecified type of upper airway infection (J06.9, 5.5%), throat pain (R07.0, 5.4%), and acute bronchitis (J20.9, 3.5%).

Pediatric and adolescent patients with airway infections or signs and symptoms thereof were significantly younger (mean 6.66 [SD 5.26] vs. 8.17 [SD 5.69] years, *p* < 0.001) and sought more often on-site medical attention (mean 3.02 [SD 2.84] vs. 1.67 [SD 1.53] visits per patient, *p* < 0.001) than pediatric patients without such a diagnosis (Figure 2B,C).

Also the subgroup of underage patients with acute respiratory infections with fever were younger than pediatric patients without such a diagnosis (mean age 4.84 years [SD 4.14] vs. 7.70 [SD 5.64] years, *p* < 0.001). The proportion of consultations due to acute airway infections or signs and symptoms thereof amongst all medical encounters per months differed between summer and winter with highest rates in December (46.7%) and lowest in August (23.3%).

Next, we performed a logistic regression model with sex, patients’ age group, region of origin, reception facility, residential density (as % of the occupancy in each facility), calendar month, and refugee category as independent variables. Robust standard errors of the regression coefficients were calculated through the use of GEE. In this model, age group (*p* < 0.001), calendar month (*p* = 0.001), refugee category (*p* < 0.001), and residential density (*p* = 0.012) had significant impact on the occurrence of cases with acute respiratory symptoms. In the extensions of this model (sensitivity analyses), we also considered two-way interactions between all significantly tested factors (i.e., age & residential density, age & calendar week, age & refugee category, calendar month & refugee category, residential density & refugee category). However, none of the tested interactions were statistically significant.

Compared to toddlers (<1 years of age), the odds ratio (OR) of young infants (1–3 years) and preschool children (4–6) years to suffer from airway infections or signs thereof was significantly increased (OR 1.51 and 1.52, respectively, both *p* < 0.001), and occupancy of 80% or higher of the reception facility was associated with a similarly increased rate of airway infections (OR of 1.64, *p* < 0.010 as compared to the reference group with 21–40% occupation, Table 1). The strongest influence on airway infections, however, was observed for month of the year with a considerable decrease over summer time (e.g., with an OR of 0.39 in August compared to reference month January, *p* < 0.001) and refugee category (OR 2.55 in refugees officially resettled by the UNHCR as compared to asylum seekers without this status, *p* < 0.001, Table 1).

## 4. Discussion

In our analysis based on a large dataset obtained shortly before the onset of the COVID-19 pandemic, we show that airway infections are a leading cause for acute morbidity in underage refugees, and that the rate of medical consultations due to airway infections is influenced by age, time of the year, residential density, and resettlement status. Data on the frequency and distribution of airway infections amongst underage migrants is scare, and we believe our analysis may help to better estimate the risk of respiratory infection spread amongst the large number of migrants currently living at reception centers or in camps around the world.

An analysis from Switzerland on the medical needs of *n* = 105 recently arrived refugee children that needed hospitalization showed that infections, particularly those affecting the airways, were the most common reason for admission [5].

Our findings are not entirely surprising given that respiratory infections are one of the leading causes for acute morbidity amongst resident German pediatric patients as well, and that their rate increases during the colder season [12,13]. An increased spread of communicable diseases at refugee centers and camps with high residential density has been reported [6,14].

The especially high rate of medical consultations due to airway infections in resettled refugees is in line with previous reports on the overall increased morbidity this subpopulation and may be in part explained by the fact that these refugees are often exposed to particularly crowded living circumstances [9,15]. Resettlement refugees go through the UNHCR refugee-status determination process and are therefore often times exposed to communal living circumstances in refugee camps or similar institutions and communal travel prior to coming to their country of destination which may put them at particular risk for communicable diseases.

Our analysis has several limitations. Firstly, due to the cross-sectional design of the present study we cannot attribute causality to the associations between any exposures and the frequency of airway infections in the study population. Although the demographic characteristics of our cohorts are representative for a large proportion of current migrants [8,9], they only represent a small sample of refugees worldwide. We classified diagnoses post hoc, possibly introducing a categorization bias. We could not normalize consultation frequencies to duration of stay in the reception centers as this information was not collected at the Friedland site. Since relatively soft definitions were used and airway infections are a rather broad diagnosis with different types of clinical manifestations, the frequency of airway infections may have been missed, underestimated, or misclassified. Although adjustments were made using a regression model, there might have been other unmeasured confounders. For example, nutritional status, past medical history and medication of your study participants might have affected the primary outcome. In addition, we could not, for example, adjust for family constellations as entities because we lacked this information.

However, our study shows with present novel, current, and comprehensive data based on a large group of underage refugees, that even in a western country with relatively well funded immigration programs, factors such as residential density significantly impact the rate of respiratory infections. As such, it sheds light on a central issue in migrant healthcare. Infections are a leading cause for migrant health concerns [16], and underage refugees have historically been a particularly vulnerable population and remain to be so in the current refugee crisis history [1,2,3,4]. In spite of this, data on their health concerns is scarce [5]. Our analysis illustrates the substantial contribution of airway infections to acute morbidity in underage refugees.

This may be particularly relevant during the current pandemic. COVID-19 impacts global economics, nutrition and health in merely unprecedented fashion [17,18]. There is an urgent need to enforce the COVID-19 response for the most vulnerable populations this humanitarian crisis [1,7], and migrating children may belong to the least fortunate in this situation. They are impacted by the pandemic with impaired education, healthcare, and malnutrition [19,20]. Importantly, the contribution of children and adolescents to the spread of the current pandemic is a matter of brisk debate [21], and governments all over the world are challenged to balance the uncertainty and risks of reopening places of communal encounter for children against the clear harms associated with prolonged closure [22]. The majority of severe acute respiratory syndrome coronavirus 2 (SARS-CoV-2) infections occur within joint housing [23,24]. Our data support the notion that the spread of airway infections is a particular problem when accommodating children and adolescents in refugee reception centers.

## 5. Conclusions

Our analysis shows that respiratory infections are a leading cause for acute morbidity amongst young refugees and that their frequency is influenced age, season, status, and residential density. Our data emphasizes the particular need to protect children from contracting airway infections in refugee centers and camps around the world. This could help to reduce morbidity and mortality in the vulnerable population of refugees and may also influence the spread of COVID-19 during the current pandemic.

## Figures and Tables

**Table 1:** Factors influencing the rate onsite consultations of airway infections of signs and symptoms thereof.

Characteristic		All Cases (*n* = 4289)	Cases with Acute Respiratory Symptoms or Signs Thereof (*n* = 1649)			
					Multivariable Analysis	
		*n* (%)	*n* (%)	% Ratio	OR (95% CI)	sig.
Sex *	**male**	2239 (52.2)	863 (38.5)	52.3	1.07 (0.94–1.22)	0.312
**Age (years)**	**<1**	521 (12.1)	168 (35.3)	10.2	reference cat.	n.a.
	**1 to 3**	1121 (26.1)	492 (47.5)	29.8	1.51 (1.17–1.95)	0.001
	**4 to 6**	842 (19.6)	375 (47.4)	22.7	1.52 (1.18–1.98)	0.001
	**7 to 12**	997 (23.2)	362 (38.9)	22.0	1.04 (0.80–1.34)	0.780
	**13 to 15**	360 (8.4)	120 (34.9)	7.3	0.98 (0.71–1.36)	0.919
	**16 to 18**	448 (10.4)	132 (31.3)	8.0	0.78 (0.58–1.07)	0.119
**Region of origin #**	**Europe & Central Asia**	398 (9.4)	134 (35.1)	8.2	reference cat.	n.a.
	**Latin America & Caribbean**	9 (0.2)	3 (33.3)	0.2	0.73 (0.27–2.03)	0.553
	**Middle East & North Africa**	2688 (63.2)	1106 (44)	67.7	1.03 (0.76–1.40)	0.839
	**South Asia**	965 (22.7)	327 (37.2)	20.0	1.08 (0.78–1.50)	0.648
	**Sub-Saharan Africa**	128 (3)	36 (29.8)	2.2	0.79 (0.47–1.32)	0.368
	**unknown/stateless**	63 (1.5)	26 (44.8)	1.6	1.07 (0.61–1.88)	0.813
**Reception facility**	**Celle**	2488 (58)	921 (40.4)	55.9	reference cat.	n.a.
	**Friedland**	1801 (42)	728 (42.4)	44.1	0.90 (0.69–1.18)	0.458
**Residential density** **(in % occupancy)**	**≤20%**	175 (4.1)	59 (36.0)	3.6	1.48 (0.96–2.28)	0.077
	**21–40%**	925 (21.6)	285 (33.8)	17.3	reference cat.	n.a.
	**41–60%**	1454 (33.9)	604 (43.6)	36.6	1.12 (0.91–1.37)	0.305
	**61–80%**	1414 (33.0)	557 (42.7)	33.8	1.02 (0.77–1.35)	0.876
	**≥80%**	321 (7.5)	144 (47.5)	8.7	1.64 (1.12–2.39)	0.010
**Calendar month**	**January**	708 (16.5)	280 (43)	17.0	reference cat.	n.a.
	**February**	638 (14.9)	260 (43.9)	15.8	1.00 (0.79–1.27)	0.999
	**March**	445 (10.4)	165 (39)	10.0	0.71 (0.50–1.02)	0.060
	**April**	314 (7.3)	86 (31.4)	5.2	0.57 (0.38–0.86)	0.008
	**May**	186 (4.3)	66 (38.2)	4.0	0.50 (0.31–0.82)	0.006
	**June**	73 (1.7)	31 (44.9)	1.9	0.67 (0.37–1.22)	0.191
	**July**	80 (1.9)	21 (27.6)	1.3	0.39 (0.20–0.76)	0.005
	**August**	154 (3.6)	35 (23.3)	2.1	0.39 (0.24–0.64)	<0.001
	**September**	388 (9)	156 (42.1)	9.5	0.80 (0.58–1.10)	0.162
	**October**	468 (10.9)	196 (44.4)	11.9	0.79 (0.57–1.08)	0.139
	**November**	256 (6)	99 (42.5)	6.0	0.80 (0.54–1.18)	0.255
	**December**	579 (13.5)	254 (46.7)	15.4	1.01 (0.73–1.40)	0.955
**Refugee category**	**Asylum seeker**	3541 (82.6)	1244 (37.9)	75.4	reference cat.	n.a.
	**Resettlement refugee**	748 (17.4)	406 (56.3)	24.6	2.55 (1.94–3.35)	<0.001

OR: odds ratio, cat.: category, n.a.: not applicable, * missing *n* = 1; # missing *n* = 37, *n* = 1 case with acute respiratory symptoms with a patient from East Asia & Pacific region.

## Data Availability

The dataset necessary to interpret, replicate and build upon the findings reported in the article will be made available on reasonable request and can be obtained by contacting the corresponding author.

## Supplementary Materials

The following are available online at https://www.mdpi.com/1660-4601/17/18/6823/s1, Table S1: Characteristics of underage refugee patients.

## Abbreviations

CI	confidence interval
COVID-19	coronavirus disease 2019
GEE	generalized estimating equations
UNHCR	United Nations High Commissioner for Refugees
ICD-10	International Statistical Classification of Diseases and Related Health Problems
OR	odds ratio
SD	standard deviation
SARS-CoV-2	severe acute respiratory syndrome coronavirus 2
