# Supplementary material for: Factors Influencing the Frequency of Airway Infections in Underage Refugees: A Retrospective, Cross Sectional Study
Source: Int J Environ Res Public Health. 2020 Sep 18;17(18):6823. doi: 10.3390/ijerph17186823 (PMC7557950; doi:10.3390/ijerph17186823)
Supplement: Supplementary file 1 [file ijerph-17-06823-s001.pdf]

Supplementary Materials:

Supplementary Table 1: Characteristics of underage refugee patients.

Characteristics	n (%)
**Total pediatric patients**	**1,957 (100)**
Celle	779 (39.8)
Friedland	1,178 (60.2)
**Pediatric patient age (in years)**	
Mean	7.34 ± 5.506
Median	6
Range	0-18
**Pediatric patients regions of origin (missing n=34)**	
East Asia & Pacific	1 (0.1)
Europe & Central Asia	207 (10.6)
Latin America & Caribbean	6 (0.3)
Middle East & North Africa	1,301 (66.5)
South Asia	296 (15.1)
Sub-Saharan Africa	66 (3.4)
Unknown / stateless	46 (2.4)
**Pediatric patients countries of origin (missing n=36)**	
Syria	923 (47.2)
Iraq	293 (15.0)
Afghanistan	289 (14.8)
Georgia	60 (3.1)
other	356 (19.9)
